# Liver uptake of gold nanoparticles after intraperitoneal administration in vivo: A fluorescence study

**DOI:** 10.1186/1476-511X-10-195

**Published:** 2011-10-31

**Authors:** Mohamed Anwar K Abdelhalim, Mohsen Mahmoud Mady

**Affiliations:** 1 Department of Physics and Astronomy, College of Science, King Saud University, P.O. 2455, Riyadth 11451, Saudi Arabia; 2 Biophysics Department, Faculty of Science, Cairo University, 12613 Giza, Egypt

**Keywords:** gold nanoparticles, sizes, time-dependent effects, liver tissue, fluorescence spectroscopy

## Abstract

**Background:**

One particularly exciting field of research involves the use of gold nanoparticles (GNPs) in the detection and treatment of cancer cells in the liver. The detection and treatment of cancer is an area in which the light absorption and emission characteristics of GNPs have become useful. Currently, there are no data available regarding the fluorescence spectra or in vivo accumulation of nanoparticles (NPs) in rat liver after repeated administration. In an attempt to characterise the potential toxicity or hazards of GNPs in therapeutic or diagnostic use, the present study measured fluorescence spectra, bioaccumulation and toxic effects of GNPs at 3 and 7 days following intraperitoneal administration of a 50 μl/day dose of 10, 20 or 50 nm GNPs in rats.

**Methods:**

The experimental rats were divided into one normal group (Ng) and six experimental groups (G1A, G1B, G2A, G2B, G3A and G3B; G1: 20 nm; G2: 10 nm; G3: 50 nm; A: infusion of GNPs for 3 days; B: infusion of GNPs for 7 days). A 50 μl dose of GNPs (0.1% Au by volume) was administered to the animals via intraperitoneal injection, and fluorescence measurements were used to identify the toxicity and tissue distribution of GNPs in vivo. Seventy healthy male Wistar-Kyoto rats were exposed to GNPs, and tissue distribution and toxicity were evaluated after 3 or 7 days of repeated exposure.

**Results:**

After administration of 10 and 20 nm GNPs into the experimental rats, two fluorescence peaks were observed at 438 nm and 487 nm in the digested liver tissue. The fluorescence intensity for 10 and 20 nm GNPs (both first and second peaks) increased with the infusion time of GNPs in test rats compared to normal rats. The position of the first peak was similar for G1A, G2A, G1B, G2B, G3B and the normal (438 nm); that for G3A was shifted to a longer wavelength (444 nm) compared to the normal. The position of the second peak was similar for G1A, G1B, G2A, G2B and the control (487 nm), while it was shifted to a shorter wavelength for G3A (483 nm) and G3B (483 nm). The fluorescence intensity of the first and second peaks increased for G1A, G2A, G1B and G2B, while it decreased for G3A and G3B compared to the control.

**Conclusions:**

The fluorescence intensity of GNPs varied with the number, size and shape of particles and with the ratio of surface area to volume in a given sample. Fluorescence intensity changes during infusion depended on the size and shape of GNPs, with smaller particles experiencing larger changes during the infusion time in addition to the quenching produced by the larger GNPs. It is likely that smaller particles, which have a much higher ratio of surface area to volume compared to larger particles, are more prone to aggregation and surface interaction with biological components. This study suggests that fluorescence intensity can be used to evaluate bioaccumulation and the toxicity of gold nanoparticles in rats.

## Introduction

Nanoparticles (NPs) offer great promise for biomedical applications, particularly in pharmaceutical delivery and novel diagnostic and therapeutic methods [1].

Despite the many potential therapeutic benefits of nanoparticles, some studies indicate that certain nanoparticles, due to their small size and unique physical properties, may cause adverse effects [2,3]. The size, surface area and dosage of particles all appear to play important roles in mediating nanoparticle toxicity. It has been proposed, for example, that the size of nanoparticles influences their adhesion to and interaction with living cells [4].

The small size of NPs may enable interactions with specific biological targets. Furthermore, metallic NPs can be made to exhibit a resonant response to a time-dependent magnetic field, producing a potentially useful energy transfer to the particles [5,6]. Therefore, such particles have been used as hyperthermic agents that deliver lethal amounts of thermal energy to targets such as tumours [7-9].

Gold NPs (GNPs) show several features that make them well suited for biomedical applications, including straightforward synthesis, stability, and the potential for surface modification with active biological molecules such as peptides or proteins [5].

Semmler-Behnke et al. observed that a considerable percentage of 18 nm GNPs is removed from the blood and trapped predominantly in the liver and spleen [10].

Fluorescence is the emission of light by a substance following absorption of light or other electromagnetic radiation of a different wavelength. In most cases, the emitted light has a longer wavelength, and therefore lower energy, than the absorbed radiation. However, when the absorbed electromagnetic radiation is intense, it is possible for one molecule to absorb two photons; this two-photon absorption can lead to emission of radiation having a shorter wavelength than the absorbed radiation.

The origin of the unique optical properties of GNPs is a phenomenon known as surface plasmon resonance (SPR). The exposure of nanoparticles to electromagnetic radiation of a wavelength much smaller than the GNP diameter induces coherent, resonant oscillations of the conduction-band electrons across the nanoparticles. These oscillations are known as the SPR, which lies in the visible frequency range and results in strong optical absorbance and scattering by the GNPs [11,12]. This phenomenon has been exploited in many applications of GNPs, such as Raman sensors [13], photocatalysts [14], and photoelectrochemical materials [15,16]. In the bioscience and medical fields, GNPs are used as immunostaining labels for electron microscopy and as chromophores for immunoreactions and nucleic acid hybridisation [17,18].

Despite these many useful applications of NPs, numerous studies have shown that exposure to smaller-sized particles produces greater inflammatory and cytotoxic responses compared to exposures to larger-sized particles at the same mass concentration [19]. It is believed that smaller-sized particles are more reactive with biological components and produce adverse effects due to the large surface area and large number of particles in a nanoparticle preparation compared to a larger-particle preparation [20].

The toxicity of NPs is thought to depend on the size, surface area, composition, and shape of the nanomaterial. Particle size plays a role in how the body responds to, distributes, and eliminates materials [14,15]; it can also affect the mode of endocytosis and cellular uptake and the efficiency of particle processing in the endocytic pathway [21,22]. Therefore, for applications of GNPs in therapy and drug delivery, it is necessary to know the bioaccumulation and local or systemic toxicity associated with the nanoparticles.

The particle-size-dependent organ distribution of GNPs has been studied in vivo [23-26]. Hyllier and Albertch showed that orally administered GNPs appeared in various tissues in mice, and that smaller particles were more readily absorbed and more widely distributed in the body than were larger particles [23]. In most studies, systemically administered NPs were primarily taken up by the liver and spleen, with lesser quantities taken up by the lung, kidney, heart, and brain after a single administration. However, little is known about the biodistribution, accumulation and toxicity of GNPs after repeated administration.

GNPs can be used in various biomedical applications; however, very little is known about the size, dose and exposure dependence of in vivo nanoparticle processing kinetics. Furthermore, there are currently no data available regarding the fluorescence spectra and accumulation of nanoparticles (NPs) in rat liver following repeated in vivo administration.

## Materials and methods

### Gold nanoparticles (GNPs)

GNPs of different sizes (10, 20 and 50 nm; products MKN-Au-010, MKN-Au-020 and MKN-Au-050, Canada, respectively) were purchased. All GNPs used in this study were in aqueous solution at a concentration of 0.01%. The mean size and morphology of these GNPs were evaluated from transmission electron microscope (TEM) images. The high electron density of gold makes this method particularly suitable for evaluating the homogeneity of the nanoparticles in terms of shape and size.

### Animals

Healthy male Wistar-Kyoto rats were obtained from the Laboratory Animal Centre (College of Pharmacy, King Saud University). Rats of age 8-12 weeks (approximately 250 g body weight) were housed in pairs in humidity- and temperature-controlled ventilated cages on a 12 h day/night cycle. A conventional rodent diet and water were provided. Forty rats were individually caged and divided into a control group (NG: n = 10), group 1 (A: infusion of 20 nm GNPs for 3 days; n = 5; B: infusion of 20 nm GNPs for 7 days; n = 5), group 2 (A: infusion of 10 nm GNPs for 3 days; n = 5; B: infusion of 10 nm GNPs for 7 days; n = 5) and group 3 (A: infusion of 50 nm GNPs for 3 days; n = 5; B: infusion of 50 nm GNPs for 7 days; n = 5). In addition, histological investigations of 10 rats were performed in support of this study. Doses (50 μl) of 10, 20 or 50 nm GNPs in aqueous solution were administered to the animals via intraperitoneal injection every day for 3 or 7 days. The rats were anesthetised by inhalation of 5% isoflurane until muscular tonus relaxed. Blood and liver tissue were collected from each rat. A blood sample of approximately 1 ml was obtained from each rat via venepuncture of an antecubital vein; the blood was mixed with 0.8 ml of heparin to prevent coagulation. In order to assess tissue uptake, as much blood as possible was collected from the rats to maximise residual blood drainage from the organs. All experiments were conducted in accordance with guidelines approved by the King Saud University Local Animal Care and Use Committee.

### Digestion of liver tissue samples

Liver tissue samples were wet-digested with nitric acid and stored as acidic digest solutions for analysis by fluorescence spectroscopy. The liver tissue was first freeze dried to minimise analyse loss and to facilitate subsequent sample preparation steps. The tissue was then homogenised to a fine powder by ball-milling in plastic containers. Approximately 0.20-0.25 g of powdered tissue was weighed into a Teflon reaction vessel and 3 ml of HNO_3 _was added. The closed reaction vessel was heated in a 130°C oven until digestion was completed. Samples were then diluted to a final volume of 20 ml with quartz-distilled water and stored in 1 oz. polyethylene bottles for subsequent fluorescence spectroscopy analysis.

### Fluorescence spectroscopy

Fluorescence spectra of liver tissue containing GNPs of different sizes (10, 20 or 50 nm) were obtained using a FluoroMax-2 spectrofluorometer (JOBIAN YVON-SPEX, Instruments S. A., Inc., France). Fluorescence measurements were made over the wavelength range 250-700 nm using 1 cm path length quartz cuvettes, which were cleaned before each use by sonicating for 5 min in deionised water and then rinsing with deionised water.

## Results and discussion

### Size and morphology of different GNPs

**Figure 1:**
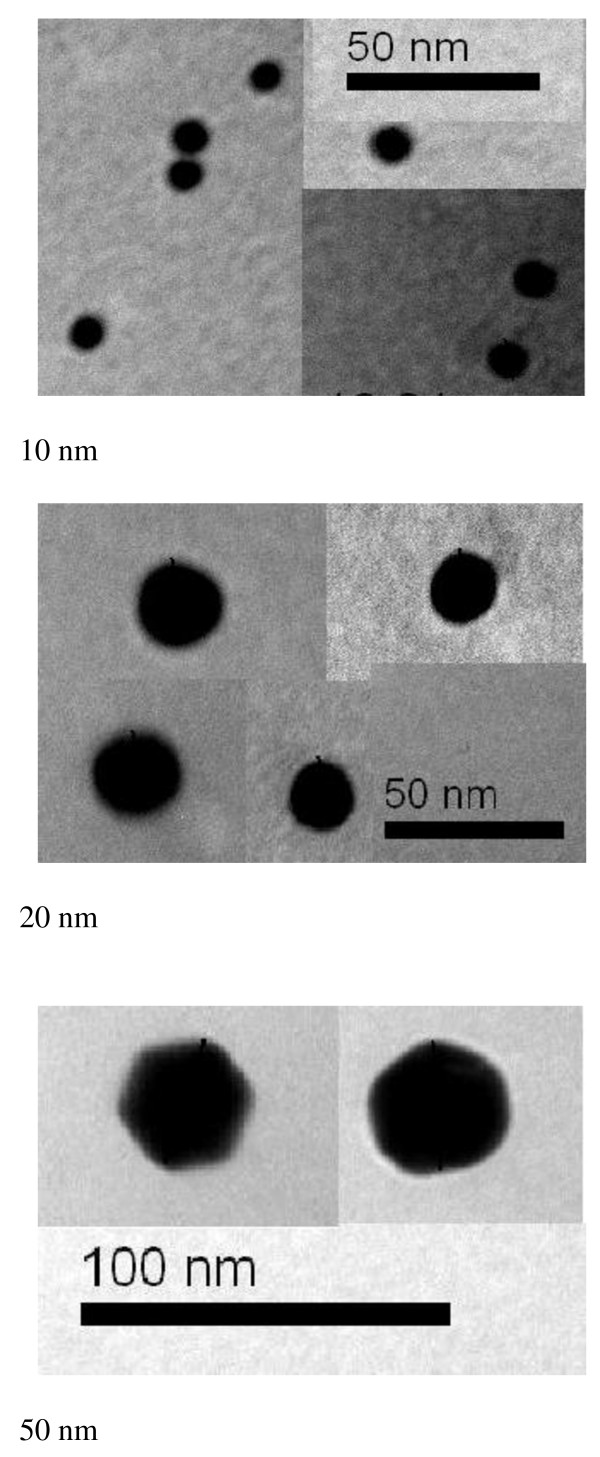
**TEM images for different GNP samples**.

The 10 and 20 nm GNPs showed spherical morphology with a narrow particle size distribution when dispersed in solution. The mean size for these GNPs was calculated from the TEM images. The mean measured size was 9.45 ± 1.33 nm for 10 nm GNPs and 20.18 ± 1.80 nm for 20 nm GNPs. GNPs of 50 nm diameters, in contrast, were not spherical but hexagonal in TEM images, as shown in Figure 1. The high electron density and homogeneous shape and size of the GNPs make them highly conspicuous under the TEM.

The maximum of the surface plasmon band (SPB) of GNPs in solution was shifted from 517 nm to 532 nm when the GNP size changed from 10 nm to 50 nm. This effect was attributed to the surface plasmon oscillation of free electrons. After injecting 20 nm GNPs into the experimental rats, we observed two fluorescence peaks at 438 nm and 487 nm in the digested liver tissue.

**Figure 2:**
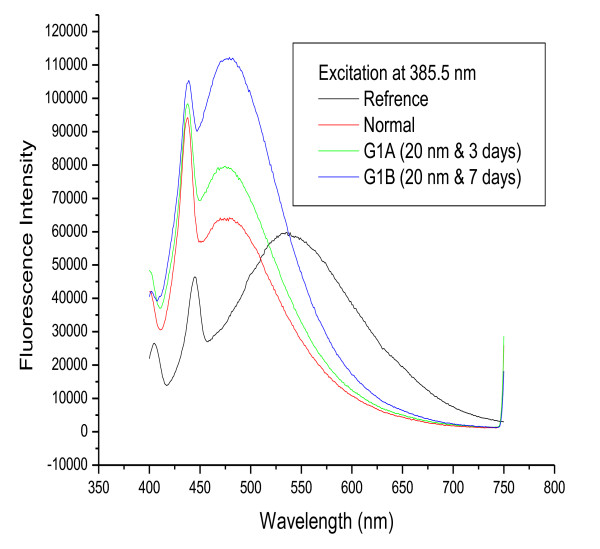
**The fluorescence emission peak intensity after infusion periods of 3 and 7 days for 20 nm GNPs**.

**Figure 3:**
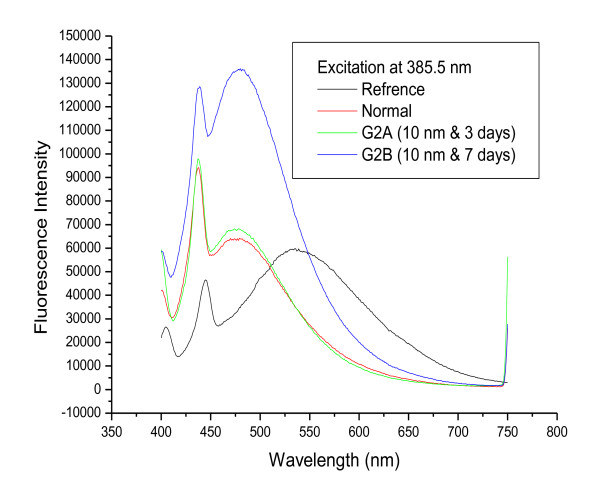
**The fluorescence emission peak intensity after infusion periods of 3 and 7 days for 10 nm GNPs**.

Figures 2 and 3 show fluorescence spectra for 20 and 10 nm GNPs, respectively, following infusion periods of 3 and 7 days. The results show that the positions of the first and second peaks were nearly the same for G1A, G1B, G2A and G2B compared with the control while the fluorescence intensity for these peaks (both first and second) increased with increasing infusion period compared to the control.

**Figure 4:**
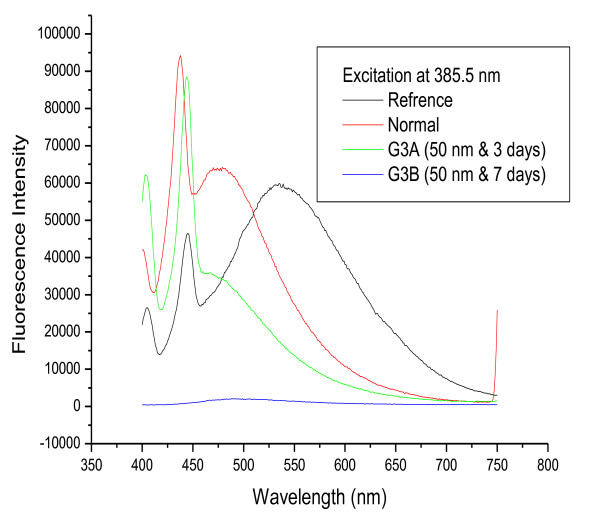
**The fluorescence emission peak intensity after infusion periods of 3 and 7 days for 50 nm GNPs**.

Figure 4 shows fluorescence spectra for 50 nm GNPs following infusion periods of 3 and 7 days. The position of the first peak shifted to a longer wavelength (first peak: 444 nm) and the second to a shorter wavelength (second peak: 483 nm) for G3A compared to the normal. However, the position of the first peak was the same as control for G3B, and the second peak shifted to a shorter wavelength (483 nm) compared to the normal. The fluorescence intensity of GNPs was lower for G3A (both first and second peaks) and much lower for G3B (both first and second peaks) compared to the control. This result may be attributed to any of three factors: 1) formation of a strong ground state complex between serum albumins and gold nanoparticles (static quenching); 2) differences in the physical and chemical properties of nanoparticles of different size and shape (as noted above, most 50 nm GNPs were hexagonal, and nanoparticle properties are highly size- and shape-dependent); or 3) faster uptake of and clearance by liver macrophages of the 50 nm GNPs compared to the other nanoparticles.

**Figure 5:**
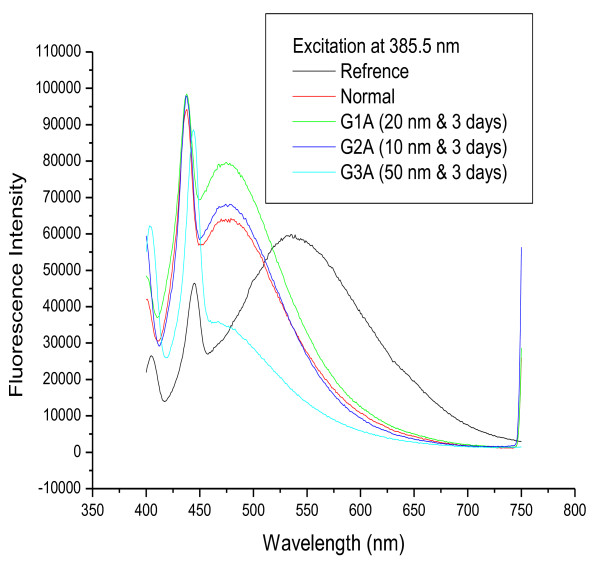
**The fluorescence emission peak intensity after infusion period of 3 days for 10, 20 and 50 nm GNPs**.

**Figure 6:**
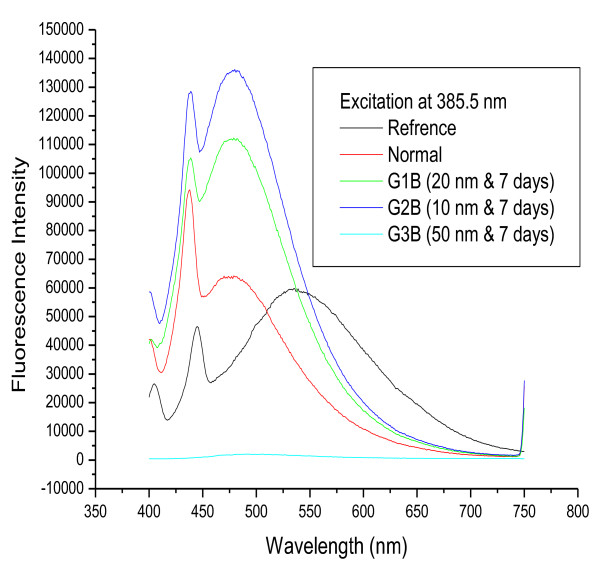
**The fluorescence emission peak intensity after infusion period of 7 days for 10, 20 and 50 nm GNPs**.

Figures 5 and 6 show fluorescence spectra for 10, 20 and 50 nm GNPs following infusion periods of 3 and 7 days, respectively.

Figure 5 shows the effect of particle size on GNP fluorescence spectra after an infusion period of 3 days (G1A, G2A and G3A). The first peak appears at a longer wavelength for G3A (444 nm) compared to the other samples, in which the first peak is at nearly the same position as the normal. The second peak appears at nearly the same position for G1A, G2A and the normal, while that of G3A is shifted to shorter wavelength (483 nm). The intensity of the two peaks was higher for G1A and G2A compared to the normal, while that in the G3A spectrum was lower.

Figure 6 shows the results of the same experiment after an infusion period of 7 days. The position of the first peak is nearly the same for G1B, G2B, G3B and the normal, while the position of the second peak is nearly the same for G1B, G2B and the normal, while the G3B peak is shifted to a shorter wavelength (483 nm). The intensity of both peaks was higher for G1B and G2B than for the normal, while the intensity of these peaks in the G3B spectrum was lower.

**Figure 7:**
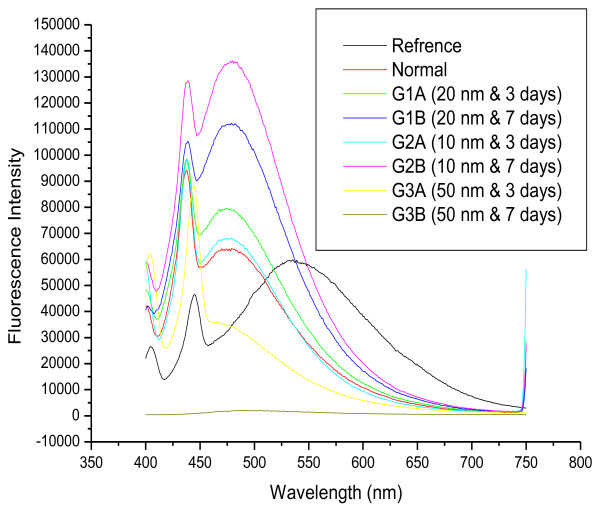
**The fluorescence emission peak intensity after infusion periods of 3 and 7 days for 10, 20 and 50 nm GNPs**.

Figure 7 shows the effect of size on the fluorescence spectra of GNPs after infusion periods of 3 days (G1A, G2A and G3A) and 7 days (G1B, G2B and G3B). The first peak is shifted to a longer wavelength for G3A (444 nm) but is nearly the same for all other samples compared to the normal. The second peak is at nearly the same position for G1A, G1B, G2A, G2B and the normal, while it is shifted to a shorter wavelength for G3A and G3B. The intensity of both peaks is higher for G1A, G2A, G1B and G2B and lower for G3A and G3B compared to the normal.

Nanotechnology has recently emerged as a promising field for the treatment and diagnosis of a variety of diseases [1]. GNPs are particularly promising because of their ease of synthesis in various shapes and the potential for conjugation with peptides and proteins, which can target the GNPs to specific interaction partners [6].

The results of this study indicate that decreasing nanoparticle size, which produces an exponential increase in surface area relative to volume, may make the GNPs more self-reactive (i.e., may promote aggregation) and more prone to interactions with surrounding molecules (biological components). Moreover, increased uptake of nanoparticles may lead to accumulation in certain tissues, where the particles may interfere with critical biological functions [16,21]. We note that the rate of exocytosis of GNPs is size dependent; larger GNPs are more readily accumulated in the cell [27].

To evaluate the time dependence of GNP distribution and aggregation, we administered 50 μl of GNPs of (10, 20 and 50 nm) by daily intraperitoneal injection into rats for periods of 3 or 7 days. At these time points, the bio-distribution of the GNPs was quantitatively measured by fluorescence spectroscopy and TEM. The size of the GNPs strongly influenced the bio-distribution.

The very small size of nanoparticles imparts physical and chemical properties that are very different from those of the same material in bulk form. Nanoparticles have a larger surface area to volume ratio compared to bulk materials; they may thus exhibit an enhanced or hindered tendency to aggregate (depending on the surface chemistry), enhanced photoemission, high electrical or heat conductivity, or improved surface catalytic activity [19,21].

Because nanoparticle surfaces can interact with biological components, nanoparticles may be more reactive than larger particles toward biomolecules. It has been shown, for example, that the severity and the likelihood of inflammatory response transiently increased, within 12 h, following injection of 200 or 100 nm GNPs into experimental animals. GNPs were ultimately trapped by macrophages in the spleen and liver and remained in these tissues until 4 weeks after the single injection [17].

To evaluate the impact of particle size on tissue distribution, we injected rats with 50 μl GNPs of different sizes. The 50 nm GNPs were taken up by liver macrophages faster and to a greater extent than smaller GNPs; the particles were eliminated after uptake. This result is correlated with the inflammatory response of the liver.

The present results show that 50 nm GNPs may be cleared via urine and bile rapidly. Nanoparticles for therapeutic use need to have a long retention time in order to encounter and interact with the desired target. However, a long retention time can result in toxic effects in vivo. Thus, route and rate of nanomaterial clearance is an important issue [28,29].

Absorbed nanoparticles in the systemic circulation can be excreted through various routes, such as the kidneys or bile. Renal clearance of solid nano-sized materials is known to be influenced by particle size and surface charge [29,30]. In the present study, we monitored the fluorescence intensity and tissue distribution of three different GNP preparations of varying particle size after intravenous injection into rats over periods of 3 or 7 days. We found that GNP fluorescence intensity and liver tissue distribution varied with particle size. The fluorescence intensity for 10 and 20 nm GNPs increased as the infusion period increased from 3 to 7 days, while it decreased during this period for 50 nm GNPs. Larger-sized particles gave lower fluorescence intensity because more particles were trapped by macrophages.

All GNPs showed a propensity to accumulate in tissues following injection. The liver tissue distribution of GNPs was size dependent: the smallest particles showed the most widespread organ distribution. Smaller GNPs also showed greater accumulation in cells according to dose-metric treatment. Therefore, it should be taken into consideration that GNPs preferentially target organs with many phagocytic cells, such as kidney, spleen, lung, heart and mesenteric lymph nodes.

## Conclusions

High electron density and homogeneous shape and size make GNPs highly conspicuous in TEM images. We found that the 10 and 20 nm GNP preparations exhibit spherical morphology, while 50 nm GNPs are hexagonal.

The size (i.e., surface area to volume ratio), shape and concentration of GNPs all contributed to differences in the fluorescence intensities between GNP samples. The time dependence of GNP fluorescence spectra was also dependent on these factors; smaller particles generally exhibited higher fluorescence that increased with GNP infusion time, whereas larger GNPs exhibited lower fluorescence decreasing with infusion time.

This study suggests that fluorescence intensity may be a useful diagnostic probe for bioaccumulation and toxicity of gold nanoparticles administered to rats. Moreover, smaller particles, which have an increased ratio of surface area to volume, appear to be more reactive toward one another (resulting in aggregation) and toward biological components in the surrounding environment.

## Competing interests

The authors declare that they have no competing interests.

## Authors' contributions

MAKA and MMM have analyzed the data, interpreted and written the final draft of this manuscript. The animal model used in this study was obtained from the Laboratory Animal Center (College of Pharmacy, King Saud University, Saudi Arabia). MAKA has conceived the study and its design and obtained research grants for this study. Moreover, both authors have read and approved the final manuscript.
